# Rapid virtual training and field deployment for COVID-19 surveillance officers: experiences from Ethiopia

**DOI:** 10.11604/pamj.2022.43.23.28787

**Published:** 2022-09-15

**Authors:** Shu-Hua Wang, Getnet Yimer, Michael Bisesi, Leuel Lisawork, David Sugerman, Mikias Alayu, Mesfin Wossen, Sisay Alemayehu Abayneh, Kayleigh Gallagher, Tigist Endashaw, Hannah Kubinson, Theresa Kanter, Kathleen Gallagher, Wondwossen Gebreyes

**Affiliations:** 1 Department of Internal Medicine, The Ohio State University College of Medicine, Infectious Disease Division, Columbus Ohio, United States,; 2 Global One Health Initiative, The Ohio State University, Columbus Ohio, United States,; 3 Global One Health, The Ohio State University, Addis Ababa, Ethiopia,; 4 College of Public Health, The Ohio State University, Columbus Ohio, United States,; 5 Centers for Disease Control and Prevention, Addis Ababa, Ethiopia,; 6 Department of Veterinary Preventive Medicine, The Ohio State University, Laboratory of Infectious Diseases Molecular Epidemiology, Columbus Ohio, United States

**Keywords:** COVID-19, virtual training, public health surveillance officers, Ethiopia

## Abstract

Rapid scale-up of surveillance activities is the key to successful coronavirus disease 2019 (COVID-19) pandemic prevention and mitigation. Ethiopia did not have a sufficient number of active surveillance officers for the public health COVID-19 response. Training of surveillance officers was needed urgently to fill the gap in the workforce needed. Subject-matter experts from the United States and Ethiopia developed applicable training modules including background on severe acute respiratory syndrome coronavirus 2 (SARS-CoV-2), contact investigation, and communications. The training modules were delivered live in real-time via web-based virtual presentation. Seventy-seven health surveillance officers were hired, trained, and deployed in two weeks to assist with surveillance activities in Ethiopia. Electronic capacity building is needed in order to improve Web-based training in resource-limited settings where internet access is limited or unreliable. Web-based synchronously delivered course was an effective platform for COVID-19 surveillance training. However, strengthening public and private information technology capacity, literacy, and internet availability will improve Web-based education platforms in resource-limited countries.

## Introduction

Coronavirus disease 2019 (COVID-19) is a global pandemic that necessitates urgent public health response. Ethiopia has a critical shortage in public health workforce to prevent and mitigate the spread of the severe acute respiratory syndrome coronavirus 2 (SARS-CoV-2) and COVID-19 [1]. The Centers for Disease Control and Prevention (CDC) and the World Health Organization recommend enhanced surveillance activities to detect, isolate, contact trace, quarantine and monitor SARS-CoV-2 transmission in communities [2,3]. In response to the shortage of health surveillance workers in Ethiopia. The Ohio State University Global One Health initiative (OSU GOHI) partnered with the Ethiopia Ministry of Health (MOH), the Ethiopia Public Health Institute (EPHI), Public Health Emergency Management (PHEM) directorate, regional health offices, CDC, and the CDC Foundation to support active surveillance training and fill the gap in workforce needed. Seventy-seven surveillance officers were hired, trained virtually and deployed to nine regions to conduct active surveillance for suspect cases of COVID-19, identify potential contacts, and conduct follow-up. An expedited timeline for training and deployment was followed due to urgent needs: job posted May 8^th^-14^th^ with 1,376 applications received, interviews and hiring between June 1^st^-June 12^th^. The minimum requirement was a college degree in nursing, public health, environmental health, or doctor of medicine. Further selection was based on academic record, prior COVID-19 training, public health field experience and region of preference. A web-based training course was developed and delivered synchronously June 15^th^-17^th^ since in-person training was not possible due to travel restrictions and recommendations for physical distancing and against large gatherings. The sessions were recorded for subsequent asynchronous viewing. All staff completed training and began working in assigned regions within two weeks. With limited experience in delivering virtual training in Ethiopia, we sought to evaluate the acceptability, feasibility, and technical aspects of web-based synchronous distance learning and provide recommendations from lessons.

## Methods

Subject-matter experts from the United States and Ethiopia (EPHI, CDC Ethiopia, CDC USA, OSU, and John Snow, Inc.) developed applicable training modules including background on SARS-CoV-2, contact investigation, and communications. The training modules were delivered live in real-time via a web-based Zoom® conference using the poll and chat features for questions and answers (Q&A) [4]. Step-by-step instructions were developed and provided to program faculty and coordinators. Due to the urgency of the pandemic, it was necessary to begin training prior to the delivery of the project laptop/tablet devices with SIM cards and Wi-Fi capabilities for the training. Pre- and post-tests and post-course evaluations were conducted anonymously using online qualtrics® survey software [5]. Ethical review was waived.

## Results

A total of 77 surveillance officers were recruited for nine regions. Table 1 shows demographics and electronic sources. All participants had access to devices for the training. The three-day training met from 9: 00 AM to 5: 30 PM Ethiopian time (3 AM to 11: 30 AM Eastern Standard Time). Training consisted of didactic and demonstration sessions. Delivery issues included muting of the audience, distracting alarms when participants logged on and off, screen sharing, video links, and launching qualtrics® or Zoom polls® [3,4]. Classroom participation through the chat box or “hand raise” features for Q&A sessions was recorded. The numbers of specific topic questions for the speakers were 43, 100, and 58 on day 1, 2, and 3, respectively. Among the 65 of 77 trainees who responded to the pre-course survey, 42% had prior COVID-19 training. Attendance rates varied from 79%-93% depending on internet connectivity. Participants also completed the free online Johns Hopkins University COVID-19 contact tracing modules [6]. Improvements were seen in the pre- and post-test (Table 2). On the post-course evaluation, 94% (61/65) of respondents were satisfied with the course, although 46% (30/65) thought the course content was too challenging. Sixty eight percent 68% (44/65) found the Web-based training technically difficult. Eighty eight percent 88% (57/65) of respondents agreed that the length of the training was appropriate. Eighty three percent 83% (54/65) stated they would use the knowledge and skills gained daily.

**Table 1:** health surveillance officers demographic and information technology availability

Demographic/electronic access	N=77 (%)
Age	27 ± 4.25 years
**Gender**	
Male	67 (87%)
Female	10 (13%)
**Degree (Highest level)**	
Bachelor science	64 (83%)
Master science/Master public health	6 (8%)
Doctor of medicine	7 (9%)
**Profession**	
Physician	7 (9%)
Nurse	24 (31%)
Public health officer	37 (48%)
Environmental health officer	9 (12%)
**Electronic device used for training**	
Personal	59 (77%)
Business	18 (23%)
**Type of devices used (Multiple may apply)**	
Desktop	8 (10%)
Laptop	38 (49%)
Tablet	9 (12%)
Smartphone	22 (29%)
No device - called in	0%
**Connection (Multiple may apply)**	
Wi-Fi	51 (66%)
3/4 G network	26 (34%)
**Internet source**	
Personal	36 (47%)
Business	41 (53%)

**Table 2:** pre-and post-test comparisons

	Pre-test	Post-test	
**Degree held**	**n=47**	**n=72**	
First degree	77%	83%	
Master's degree	11%	8%	
Doctor of medicine	13%	8%	
**Years of experience**	**n=48**	**n=68**	
Less than one year	40%	38%	
One to five years	40%	31%	
More than five years	21%	31%	
	**Number of respondents**	**Number of respondents**	**Difference in percentage of correct response**
Q1. Which of the following is the importance of data gathered and analyzed from contact tracing?	n=46	n=73	93% to 99% =6%
Q2. Indicate two general modes of exposure to SARS-CoV-2	n=43	n=70	84% to 93% =9%
Q3. Indicate the category of exposure control that involves using an artificial barrier to separate contact between a person and another person, air, objects, or surfaces contaminated with SARS-CoV-2:	n=48	n=73	90% to 90% =0%
Q4. When testing for COVID-19 using a molecular test, which is the best specimen to collect?	n=48	n=73	65% to 88% = 23%
Q5. When collecting a nasopharyngeal specimen, which statements are true? select all that apply	n=46	n=72	17% to 46% = 29%
Q6. A negative PCR test means that:	n=47	n=73	68% to 73% =5%
Q7. What factors affect the laboratory's ability to get an accurate test result (check all that apply)	n=47	n=73	62% to 77% = 15%
Q12. Which one of the following is the objective of COVID-19 surveillance	n=47	n=72	94% to 99% =5%
Q8. What are the steps of an outbreak investigation?	n=45	n=73	69% to 82% = 13%
Q9. Which of the following variables should be included on a line list?	n=45	n=73	100% to 100% = 0%
Q11. Which one of the following is not close contact to COVID-19 cases	n=46	n=73	48% to 66% = 18%
Q13. Which of the following is not considered as public health emergencies in Ethiopia	n=46	n=72	24% to 83% = 59%
Q14. Which of the following is not among priority diseases under surveillance in Ethiopia?	n=46	n=72	33% to 68% = 35%
Q15. Which of the following is a critical element during risk communication? Select all that apply	n=42	n=73	48% to 59% =11%
Q16. A weakness in risk communication management in Ethiopia include which of the following? Select all that apply	n=44	n=73	20% to 43%=23%
Q17. While engaging the community which of the following ethical principles should be considered?	n=46	n=73	93% to 93% = 0%
Q18. During pandemics individual's right outweighs the benefit to the community	n=46	n=73	72% to 73% =1%
Q19. Knowledge about COVID-19 in Ethiopia very high	n=47	n=73	19% to 34%= 15%
True	19%	34%	15%
False	81%	66%	-15%
Q20. When handling COVID-19 considering engagement of the lowest administrative structure in the country is more effective than a higher level engagement	n=46	n=73	67% to 75% =8%

## Discussion

We successfully delivered an effective web-based COVID-19 training synchronously to fill a gap in the health-workforce, while overcoming some information technology (IT) challenges.

**Geographic diversity:** the number of staff for each region and percentage of total staff hired were: Afar 7 (9.1%), Amhara 8 (10.4%), Benishangul-Gumuz 8 (10.4%), Dire Dawa City 4 (5.2%), Gambela 8 (10.4% ), Harari 3 (3.9%), Oromia 9 (11.7%), Somali 8 (10.4%), Southern nations, nationalities and peoples´ 7 (9%), Tigray 6 (7.8%), and Addis Ababa City 6 (7.8%). The numbers of regional consultants were based on COVID-19 risks (availability of point of entry, community and health system vulnerability), incident rate of the hazard (attack rate and fatality rate), and health system and population capacity.

**Workforce:** in order to increase and strengthen workforce capacity, no one working for MOH, EPHI, or PHEM were recruited. This may have limited the pool of experienced applicants, as 82% had less than or equal to five years. It is unknown if this younger aged workforce had more familiarity with internet or smartphones and could better adapt to web-based training.

**Information technology platform:** Ohio State University (OSU) has experience hosting in-person training workshops and webinars in Ethiopia, but this is the first extended web-based course. Several participants experienced internet connectivity issues, especially those residing in rural areas of Somali and Gambella with low Wi-Fi strength or limited Ethernet bandwidth. Portable document format (PDF) copies of presentations and call-in phone number were provided. Regional toll-free line could be set up or the organizers could call the participants to avoid caller costs. Another issue encountered with disruptive connectivity was multiple restart logins during tests and surveys. The organizers needed to delete multiple incomplete responses. Sessions were recorded for review later than needed. One positive outcome with digital platforms was the increased classroom participation with the use of chat box or “hand raise” features for Q&A sessions. The chat box granted those individuals who may not normally speak up in class an equal voice.

**Information technology (IT) literacy:** besides internet connectivity, some participants had general issues with digital platforms such as logging onto training modules, taking surveys/poll or joining a breakout room. A simplified user guide was provided to attendees. Organizers should host an informational session prior to the start of the course. During the course, It-related questions were answered in real-time on Zoom chat and by telephone.

**Time zone difference:** Ethiopia-based faculty tailored the presentation specifically for current recommendations and practices in Ethiopia and the US-based faculty joined in the afternoon (7AM-11: 00 AM EST). The diverse faculty with expertise in COVID-19 response in Ethiopia and the US were highly valued by the participants in the survey comments.

**Education:** Ohio State University Global One Health initiative´s (OSU GOHi) key mission is to build training and education capacity. This COVID-19 training is on an OSU institutional forum for open viewing with potential to reach an expanded global audience with even greater impact. Continuing education and weekly follow-up after the initial Web-based course was provided to trainees by the OSU GOH office and regional supervisors to ensure all required topics were covered in sufficient scope and depth.

## Conclusion

Based on the post-test survey results and course reviews, our web-based synchronously delivered the course was an effective platform for COVID-19 surveillance training in a manner consistent with guidelines for physical distancing in Ethiopia. However, enhanced electronic capacity building is needed in order to improve web-based training in resource-limited settings where internet access is limited or unreliable. Strengthening public/private IT capacity, literacy, and internet availability will improve web-based education platforms. The surveillance officer trainees are currently successfully contributing to COVID-19 health bureau activities in Ethiopia.
